# A Qualitative Assessment of Return to Sport in Collegiate Athletes: Does Gender Matter?

**DOI:** 10.7759/cureus.9689

**Published:** 2020-08-12

**Authors:** Allison M Morgan, Claire E Fernandez, Michael A Terry, Vehniah Tjong

**Affiliations:** 1 Department of Orthopaedics, Northwestern University Feinberg School of Medicine, Chicago, USA

**Keywords:** gender comparison, return to sport, young athlete, female athlete, qualitative studies, rehabilitation psychology

## Abstract

Introduction: Participation of female athletes in collegiate athletics continues to rise, but there remains a significant underrepresentation of this growth in the literature and lack of knowledge regarding the impact of gender on the college athlete experience. Our goal was to explore how collegiate female and male athletes perceive and approach return to sport after orthopaedic surgery.

Methods: Semi-structured, open-ended interviews were conducted with collegiate varsity athletes from a single institution who underwent orthopaedic surgery following injury with at least two years follow-up. Athletes were asked about factors influencing recovery, rehabilitation, and their return to or retirement from sport. Codes, categories, and themes were derived within and across genders.

Results: Fifteen athletes (six females and nine males) were interviewed individually. Athletes shared similar experiences following injury, citing similar motivations driving them back to sport. Athletes stressed the importance of the athlete role to their identity regardless of gender. Our analysis revealed two gender-related challenges: male athletes commonly felt weight change was a barrier to successful recovery and often led to self-consciousness; while females expressed frustrations in lack of empathy from those they turned to for support.

Conclusion: Female and male athletes shared some common supporting and challenging factors in return to sport following orthopaedic surgery. The most important findings of the present study were the differentiated challenges male versus female athletes experienced. Female athletes found difficulty with interpersonal relationships and external support, while male athletes struggled internally with their own body image and changing self-concept. This qualitative study provides a nuanced look at the experience of varsity athletes returning to sport following surgery. An understanding of the gendered experiences of collegiate athletes is critical to ensure all athletes in this unique population are supported as they cope with injury and seek to return to sport.

## Introduction

Participation in National Collegiate Athletic Association (NCAA) sport reached an all-time high in the 2017-2018 season with nearly half a million athletes, with female athletes representing 47% of collegiate Division I athletes [1]. However, there remains significant underrepresentation of female athletes in sports literature and a need for understanding of the role of gender in sport [2-4]. Limited existing literature suggests there may be psychological differences between male and female athletes in the way they experience sport and recover from injuries. For example, female athletes have higher self-reported depression and experience less social support than male athletes [5,6]. Male and female athletes at varying levels of sport have also demonstrated differences in narrative sports experience [7], in attitudes toward risk, pain, and injury in sport [8], and in psychological response to injury [9]. Though the literature suggests there may be differences between male and female athletes in general, the topic remains underexplored in the population of collegiate athletes. This at-risk population faces unique psychological and environmental circumstances and it is crucial to better understand their experiences to provide optimal care after injury [10].

Qualitative methods are especially well suited to investigate nuances in experience including psychological and interpersonal factors. Such qualitative approaches have elucidated important factors influencing return to sport in a variety of clinical scenarios [11-13]. Sports injuries requiring surgery are a significant concern for athletes and their health care providers, and as the impact of gender is increasingly recognized in medicine and society it is crucial to understand its impact specifically on the injured collegiate athlete. This study aimed to understand the impact of gender on return to sport for collegiate athletes and their experiences recovering from orthopaedic surgery. Understanding these factors may allow for better counseling and identification of gaps in care to improve interventions for these high-level athletes.

## Materials and methods

Design

A retrospective qualitative approach was utilized consisting of semi-structured interviews. The semi-structured interview guide was developed from a review of sports psychology, gender, and qualitative sports medicine literature [5-9] (See Appendix). This guide served as a template with additional questions iteratively added based on previous interviews to capture more of participants’ perspectives and experiences. Data was analyzed using a grounded theory approach to identify themes. Design and analysis protocols were developed by the senior author who has extensive experience conducting qualitative research.

Participants

This study included current and former varsity athletes who required orthopaedic surgery during their collegiate career. All surgeries were secondary to injuries sustained during athletic activity, with a variety of athletes and injuries intentionally chosen to increase generalizability. All athletes played at a single NCAA Division I institution and were injured between 2011-2016. This time frame was chosen to ensure at least two years of follow up after injury while minimizing recall bias by excluding surgeries prior to 2011. Gender was ascertained during interviews. All athletes interviewed identified as cisgender; that is, their gender aligned with their birth sex. Furthermore, this gender aligned with the classification of the NCAA team for which they played (i.e. interviewed women’s field hockey players identified as female). Given this finding, the terms gender and sex; male and man; and female and woman will be used interchangeably throughout this manuscript. The university Institutional Review Board approved of this study.

Procedures

**Figure 1:**
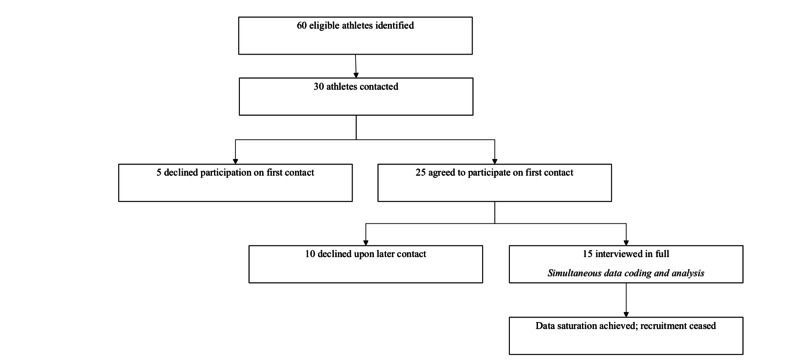
Recruitment process

Eligible participants were contacted by mail and/or email, followed by a telephone call to gauge interest, explain the study, and provide the opportunity to ask questions. Informed consent was obtained, and the rights of subjects were protected. Recruitment processes are described in Figure 1.

For the cohort of 15 athletes who ultimately participated, author A.M. conducted 30-45 minute in depth, open-ended interviews. The interviewer began with open-ended questions about the athlete’s college career to establish rapport and gather contextual information of the athlete’s experience. Using methods of active listening, the interviewer allowed the interviewees to guide the conversation, turning to the interview guide when appropriate to ensure all relevant information was solicited. Towards the end of interviews, athletes were asked explicit questions about gender’s influence on their experiences if this topic had not been spontaneously addressed. All athletes were given the opportunity to supply any other information they felt important to their experience at the conclusion of the interview and provided follow up information to contact the interviewer if further insights arose. When no new codes or themes emerged during analysis as described below, data saturation was achieved, and recruitment was concluded.

Analysis

Interviews were recorded and transcribed in their entirety by author A.M. Anonymity was preserved using alphanumeric identifiers for each participant and omission of identifying details from transcriptions. Three members of the research team (A.M., C.F., V.T.) independently coded these transcriptions in Microsoft Excel using an open coding method [14]. The team discussed coding after each interview to create a final coding schema for each interview, with disagreements resolved with discussion until consensus was achieved. As additional interviews were coded, the codebook continually was expanded and iteratively revised and each previously analyzed interview was reviewed and recoded as necessary. A single common codebook was used for all interviews of all genders. Codes were then grouped into related categories using a grounded theory approach [14]. The method used to compare male and female groups was developed based on literature review and previously published approaches as summarized by Lindsay [15]. The frequency of each codes’ use by gender was identified and compared across genders as an initial means of identifying areas of potential gender differences. As the codebook was generated throughout data analysis, codes were sorted into iteratively revised categories. These categories were compared and contrasted between gender groups and connections explored to generate overarching themes.

## Results

In total, 15 athletes were interviewed, six women (40%) and nine men (60%), representing five sports. All athletes underwent at least one surgery for lower extremity injury with the exception of two who underwent shoulder labrum repair (one woman and one man). Demographic data is presented in Table 1.

**Table 1:** Demographics and details on athletic careers “Redshirting” refers to an athlete remaining out of competition for one year in order to extend National Collegiate Athletic Association (NCAA) eligibility.

	Female	Male
Lacrosse	3	0
Field Hockey	1	0
Tennis	1	0
Basketball	1	0
Football	0	6
Soccer	0	3
Underwent multiple surgeries during college career	67% (4/6)	67% (6/9)
Redshirted*	50% (3/6)	56% (5/9)
Completed college career	83% (5/6)	78% (7/9)
Aspired for professional career	50% (3/6)	56% (5/9)
Played at professional level	0% (0/6)	11% (1/9)

**Figure 2:**
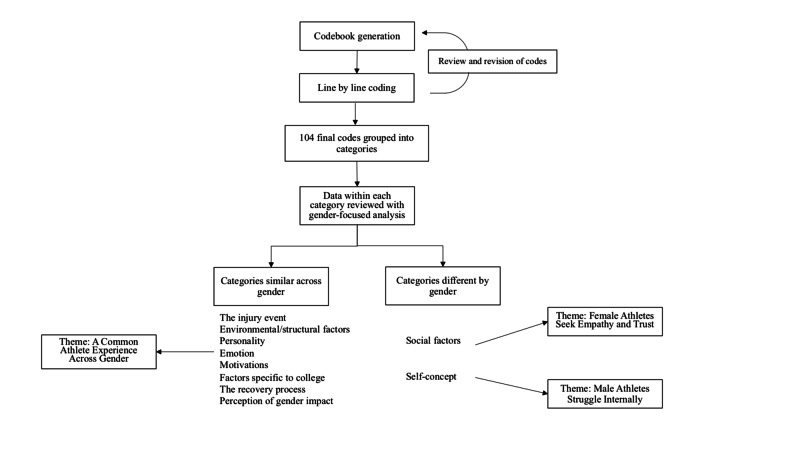
Coding and thematic analysis

Interview analysis resulted in 104 unique codes which were grouped into ten categories compared across gender; the process of data coding and thematic extraction is outlined in Figure 2.

Ultimately, three themes emerged: athletes share many commonalities in experience regardless of gender, however, female athletes tended to be frustrated by interpersonal interactions while male athletes more often struggled most with their internal sense of self. 

A common athlete experience across gender

Although this study categorizes athletes as either male or female, all participants identified themselves above all else as simply ‘athlete.’ This role motivated participants through rehabilitation and was so key to sense of self that few athletes even considered retirement after experiencing injury: ‘I’ve been doing this [sport] my whole life; it is who I am’ (F1), ‘to say goodbye to [sport] … was something that I was not ready to accept or even crossed my mind to be honest’ (M1). Conversely, athletes felt challenged when this athlete role was threatened: ‘As athletes we generally find our identity, like who we are, in our sport, so when you’re injured, and your sport’s taken away from you, it can be crushing’ (M7). Athletes also described difficulty keeping up with the extremely time-consuming process of recovery and its mental and physical tolls: ‘You don’t see progress day in day out, it takes weeks…it can be hard to stay motivated’ (M5), ‘I was really, really tired all of the time and I was really stressed’ (F4), ‘I know they say school’s supposed to be a priority but it’s not like that … sleep was affected and schoolwork was affected’ (M2). 

In coping with challenges, athletes cited various interpersonal drivers including athletic trainers and teammates: ‘I care a lot about not letting my teammates down and being there and helping how I could’ (F4), ‘the other injured guys .... can help you motivate, like we’re all in this bad situation together, we can get out of it’ (M2). Notably, players who graduated or retired felt they did not have the same level of social support, making the physical rehabilitation and the psychological adjustment more difficult: ‘it wasn’t the same athlete environment where I saw lots of support … the environment wasn’t the same community that I relied on in college for my first surgery’ (F5), ‘Once you’re done playing the sport, you’re done… there wasn’t much support there, which isn’t really anybody’s fault, it’s just the reality of it all’ (M4).

When asked if gender impacted recovery or return to sport, most athletes did not perceive differences. Instead, they offered similarities, particularly this shared experience of injured athlete: ‘I wouldn’t see [female student athletes] as any different in terms of their recovery process, in terms of the pressures and demands on them’ (M9); the athlete identity was ‘similar for both males and females-especially top athletes. We have the same drive’ (F1). Overall, male and female athletes perceived their experiences with sport and injury to be more similar than different, experiencing common drivers and difficulties in pursuit to reclaim their athlete identities.

Female athletes seek empathy and trust

Athletes of both genders described the importance of social support in their recovery process. However, female athletes specifically noted insight into their experience was a crucial factor in making a social supporter effective. As one athlete explained, the psychologist most impactful in her recovery was helpful because ‘she just kind of got me’ (F2). Female athletes experienced difficulties in their recovery process when they did not feel this understanding from the training staff, teammates, family, or non-athlete friends and peers. They struggled with feeling ignored, that their hardships were not understood, or that their motivation to go through the arduous process of rehabilitation was questioned: ‘The hardest thing was my friends outside of my team and outside of my family because I feel like if you don’t play [my sport] you don’t really understand that it’s super demanding’ (F6); ‘Although everyone was nice and supportive, I felt like they didn’t really understand’ (F3). Interestingly, one female athlete drew parallels to male football players, explaining, ‘It’s like an NFL football player that gets concussed and can never play anymore. This is what I’m good at so it’s hard to stop… This is my passion. This is what I’ve put years of my life into, so it’s not as easy as just not playing which a lot of people don’t get’ (F1).

This lack of empathy extended further with female athletes perceiving they were not trusted by coaches and training staff while calibrating their recovery. Some females felt they were pushed too hard by coaches: ‘That was very frustrating when I would go to a practice and I would be like listen, I’m so sore, I feel like I was hit by a bus, I can’t move, and they’d be like, ‘Get on the line,’ and I was like, I am a [multiple] surgery, post-surgery person, why am I running this much?’ (F1), while others felt they were not pushed hard enough: ‘Some of the collegiate athletic trainers are a little, not wimpy, but I feel like you just could be giving more… I would do my rehab a second time or third [on my own]’ (F6). Facing these environmental challenges, our female athletes found motivations from within: ‘there were so many times were I was like I need to transfer I need to go home but that’s not the person I am, I was finishing [my career]’ (F1), ‘I made a promise to myself that I would keep playing’ (F3). Throughout their injury and recovery processes, our female athletes sought empathy and trust from those surrounding them and found their biggest challenges at the limits of that understanding.

Male athletes struggle internally

While female athletes struggled to be heard by coaches and athletic trainers, male athletes felt more confident that athletic staff listened to them and balanced rest and rehabilitation: ‘I would just be explicit and tell people like no this doesn’t feel right…. being open in communication with them, which they accepted’ (M4), ‘My coach made good decisions not to push me too hard or play me too many minutes’ (M8), ‘[my athletic trainer] knew when to push and he knew… when the body was breaking’ (M1).

With support from social relationships in place, male athletes instead felt more challenged from within: ‘it’s just like you’re battling you and your mind in recovery’ (M2). While rehabilitating from injury and returning to or retiring from sport, male athletes struggled with body image and its centrality to their sense of self. Male athletes experienced both weight gain and loss post-surgery and found this a significant barrier to return to play: ‘The hardest part for me by far… when I kinda thought maybe I’m not gonna come back, this is gonna be really tough, was getting back in shape again’ (M9), ‘It was like, oh man I got really fat’ (M1). This posed a more than a physical challenge, as male athletes felt a psychological toll in the dissonance between their physique and their identity as athlete. One male who lost significant weight explained, ‘You’re so used to being …the athlete people look upon, and the moment that you get hurt, you get kind of human in a sense, and you lose the ability to wow people based on your athletic prowess’ (M6). Male athletes whose injuries ultimately forced them to quit sport also expressed difficulties with body-identity dissonance. One male who left his sport due to concussion explained that leaving ‘was one of the biggest challenges that I’ve had in my life… because my body was completely capable of still playing’ (M4). Another who retired talked at length about adjusting to his body as non-athlete, explaining ‘it was very important to me to be able to kind of change my identity. Walking around school, you’re a fairly large person … if you’re larger than the normal student they’ll assume you’re an athlete and so I really tried hard to lose a lot of weight and look more ‘normal’ (M3). Whether striving to return to play or struggling to adjust to non-athlete life, our male athletes found the appearance of their bodies central to their self-image and psychological struggles surrounding their changing identities.

## Discussion

The most important findings of the present study were the unique challenges male and female athletes experienced, revealing the importance of internal self-conception and external supports on psychological state for our athletes during recovery. Psychological factors and readiness have previously been identified as critical in athlete’s return to sport after injury [16-21]. Importantly, the literature has previously suggested that male and female athletes do not experience those psychological aspects of injury or return from injuries in the same ways [2,19,22]. For example, female athletes have been found to be less likely to return to sport following ACL reconstruction [23] while male athletes demonstrate higher psychological readiness to do so following surgery [20,24]. Following concussion, adolescent male and female athletes recovery courses have been shown to differ in length and required treatment interventions [25]. Understanding the root of such differences and if gender truly matters is crucial. While the gap continues to narrow between male and female participation in sport, research on these potential sex and gender-based differences remains significantly lacking [3,4]. 

Our findings shed light on the challenges which may be impeding female athlete’s return to sport. Female athletes have been shown to have higher levels of social anxiety than male athletes [5] and to struggle with lack of social support throughout their athletic career [7]. Similarly, our female athletes struggled with the limits of their social support after injury, often feeling that they did not receive empathy and that their drive to play was questioned. Males did not describe such challenges with being misheard or misunderstood. This may be due to societal expectations that sport, especially at elite levels, is a predominantly masculine endeavor. Though professional male athletics remain more visible and hugely more profitable than professional women’s athletics [26], half of our female athlete cohort aspired for professional careers. Such aspirations and drive to push through physical pain and adversity may fit into societal expectations of male behavior, while females exhibiting this drive do not easily align in society’s gender schemas. Our female athlete who compared herself to an NFL football player to characterize her drive and dedication to sport provides a marked example of how gender expectations and models of behavior pervade even our female athletes themselves.

As an extension of this misunderstanding, our female athletes experienced frustrations with athletic staff not trusting them to lead their recovery. In the overarching sense of purpose and in the daily activities to achieve their goals, our cohort of female athletes felt their desires both to push their body further or to hold back were not always accepted. They often felt as though they had to work against outside forces, at times including members of their rehabilitation team, to gain autonomy over recovery. This echoes prior work demonstrating tensioned relationships between females and coaches following injury [9]. Meanwhile, males more often trusted that athletic trainers and coaches were making the right choices or were willing to follow the athlete’s lead.

With greater support surrounding their recovery process, our male athletes’ greatest challenges instead came from their own psyche. Male athletes struggled with their changing identity and physique, whether temporarily in injury or permanently when facing retirement. Though body image is often discussed in society as a female issue, male and female elite athletes alike struggle with psychological difficulties surrounding body image [27,28]. Galli and Reel have previously described the significant concerns male athletes experience with body changes [29]. Our male athletes emphasized these concerns and the centrality of physicality to sense of self. Athletic and medical staff should be cognizant of the importance of body image in male athletes following injury and in transitioning back into and out of athletics.

All athletes experienced difficulties with transitioning out of the varsity athlete role. Whether due to graduation or retirement, our collegiate athletes described how this change made the logistics of recovery more difficult and more importantly signaled a significant shift in mentality. Without the motivators and team environments previously driving them, former athletes found rehabilitation more difficult and struggled with the global change in identity that came with no longer being an athlete. College athletes are a uniquely vulnerable population and one positioned to benefit from change at an institutional level. Targeted interventions towards former players, while they remain on campus and coordination of care with new physical therapists, psychologists, etc. after graduation or retirement, may ease this tumultuous time and ensure former athletes are cared for physically and mentally.

It is important to acknowledge that much of our athletes’ experiences with injury were similar and that participants self-identified as athlete, not ‘male athlete’ or ‘female athlete.’ However, this work focuses on the differences between males and females to specifically understand gendered experiences of varsity athletes. Furthermore, though we group our athletes as male versus female, gender is in fact not a dichotomy and encompasses many aspects of one’s self-identity [30]. Due to the existing framework of separated male and female athletes in collegiate and across sport worldwide, we chose to explore gendered differences in the current sport-imposed binary [26]. Capranica et al. explain that cultural and social factors hugely impact observed differences in male and female athletic performance and experience [26]. As these societal influences change over time, so too may the experiences of male and female athletes and, importantly, those who do not identify within this gender binary at all. 

This qualitative study provides a nuanced look at both common and gendered experiences of college athletes following injury in return to sport. However, this study has several limitations. Recruitment conclusion occurred when data saturation was reached, a determination made by the researchers, and thus subject to interviewer bias. Additionally, athletes who chose to participate may experience recall or social desirability bias and thus not reflect the experience of all athletes. Variability in the actual sports played and injury experienced may introduce confounding variables, but this diversity in our sample was purposeful in order to generate themes which would be more globally applicable across athletes. Finally, because this cohort consisted of collegiate varsity athletes at a single institution, conclusions may not be applicable to athletes of all levels.

## Conclusions

Male and female collegiate athletes share similar motivations and difficulties in returning to sport following orthopaedic surgery, however, gender does influence return to sport in college athletes; female athletes often find their greatest challenge in interpersonal relationships, while the injured male athlete more often struggles internally with his own identity. Understanding the gendered experiences of athletes is critical to ensure all athletes are appropriately counseled and supported as they cope with injury, recover from orthopaedic surgery, and seek to return to sport.

## Appendices

Appendix: Semi-structured Interview Guide

Establish rapport: Tell me about your injuries as an athlete.

Open: What factors do you think were most important in your return to/retirement from sport, and why?

Additional prompts, as necessary:

·      How was your mood during your recovery progress? How did you cope?

·      How did your personality type play a role in your recovery process?

·      How did the people around you impact on your recovery?  [Prompts: Coach, teammates, athletic staff, family, friends, community]

·      Do/did you ever experience fear that you will re-injure yourself? Why or why not?

·      If not still playing sport: Why did you ultimately retire from play?

·      For athletes who experienced multiple injuries: How did your recovery process differ over subsequent injuries?

On Gender: How do you think your experience was the same or different as an athlete of the opposite sex or gender?
